# Effects of Bioconverted Guava Leaf (*Psidium guajava* L.) Extract on Skeletal Muscle Damage by Regulation of Ubiquitin–Proteasome System and Apoptosis in Type 2 Diabetic Mice

**DOI:** 10.3390/ijms26083877

**Published:** 2025-04-19

**Authors:** Heaji Lee, Bo-Gyu Jun, Su-Hyun Kim, Choong Hwan Lee, Yunsook Lim

**Affiliations:** 1 Department of Food and Nutrition, Kyung Hee University, Seoul 02447, Republic of Korea; ji3743@khu.ac.kr; 2 Department of Bioscience and Biotechnology, Konkuk University, Seoul 05029, Republic of Korea; qhrb030@naver.com (B.-G.J.); kimsuhyun2019@naver.com (S.-H.K.); chlee123@konkuk.ac.kr (C.H.L.); 3 Research Institute for Bioactive-Metabolome Network, Konkuk University, Seoul 05029, Republic of Korea

**Keywords:** type 2 diabetes mellitus, muscle atrophy, guava leaf (*Psidium guajava* L.), *Lactobacillus plantarum*, metabolites

## Abstract

Skeletal muscle atrophy is one of the serious complications of diabetes, which increases the risk of frailty, falls, and mortality. However, interventions for muscle atrophy are limited, and research is needed regarding the treatment of muscle wasting. Recently, the bioconversion of natural products by lactic acid bacteria has been highlighted as a possibility to improve the bioavailability of active ingredients. This process also produces metabolites, which are key signaling mediators for a variety of physiological functions. This study investigated the effect of bioconverted guava leaf (*Psidium guajava* L., GL) by *Lactobacillus plantarum* on hyperglycemia-induced skeletal muscle atrophy in type 2 diabetes mellites (T2DM) mice. Diabetes was induced by a high-fat diet with a two-time streptozotocin (STZ) injection (60 mg/kg BW) in male C57BL/6J mice. After diabetes was induced (a fasting blood glucose level (FBG) ≥ 300 mg/dL), the mice were administered with GL (100 mg/kg/day) or bioconverted GL (FGL) (50 mg/kg/day) by oral gavage for 14 weeks. FGL contains different substances such as hydroxyl-isocaproic acid and hydroxyl-isovaleric acid compared to GLE itself, which have potential to prevent muscle degradation in T2DM mice. GL and FGL supplementation reduced the FBG level in T2DM mice. In addition, GL and FGL supplementation enhanced muscle strength, the skeletal muscle cross-sectional area, and ameliorated ubiquitin–proteasome system (UPS)-related pathways in T2DM mice. On the other hand, GLE supplementation ameliorated glucose tolerance demonstrated by oral glucose tolerance test and enhanced insulin signaling pathway. In addition, only FGL supplementation attenuated skeletal muscle inflammation and apoptosis with an improved mammalian target of the rapamycin (mTOR)-autophagy-related pathway. Although administered at a half dose of GLE, FGL demonstrated greater efficacy in regulating the expression of these molecular markers. The result suggests that even GL itself has anti-diabetic effects, and the functionality would be enhanced by the bioconversion of GL with *L. Plantarum*, which has an additive or/and a synergistic effect. Taken together, FGL could be used as a potential nutraceutical to attenuate muscle degradation by the inhibition of inflammation, the UPS, and the apoptosis pathway.

## 1. Introduction

Type 2 diabetes mellitus (T2DM) is characterized by chronic hyperglycemia causing a lot of diabetic complications [1]. Among them, skeletal muscle atrophy is one of the serious complications of diabetes, which increases the risk of frailty, falls, and mortality [2]. T2DM induces a shift in the muscle fiber type from slow-twitch to fast-twitch, leading to energy deficiency [3]. Failure of proper glucose utilization caused by insulin resistance activates muscle proteolysis through protein degradation pathways in T2DM [2,3].

The ubiquitin–proteasome system (UPS) and apoptosis are major pathways that contribute to protein degradation, thereby promoting muscle atrophy [4]. In particular, the muscle-specific E3 ubiquitin ligases muscle RING-finger protein-1 (Murf-1) and muscle atrophy F-box (MAFbx)/Atrogin-1 are key factors that are increased in a muscle atrophic condition [4]. Previous studies showed that the expression of Murf-1 and Atrogin-1 were increased in the skeletal muscle of T2DM [5]. In addition, myostatin is known to have a crucial role for muscle wasting. Myostatin activates the translocation of foxo3a into nucleus and then increases the expression of muscle-specific E3 ligases (Atrogin-1 and Murf-1) [6]. Myostatin also induces apoptosis via the mitochondrial apoptotic pathway, leading to increased protein degradation [7].

Apoptosis is a programmed cell death that contributes to the maintenance of homeostasis [8]. The persistence of hyperglycemia stimulates the production of pro-inflammatory cytokines, which trigger apoptosis [8]. Excessive apoptosis leads to the degradation of muscle protein and atrophy of muscle fibers, resulting in muscle mass loss [8]. Previous evidence showed that chronic inflammation could activate pro-apoptotic Bax, which releases cytochrome c and subsequently activates caspase-3 [9]. In addition to apoptosis, insufficient autophagy caused by hyperglycemia causes deficiency in the removal of misfolded proteins or damaged organelles, which can contribute to muscle damage [10].

Many medical or nutritional interventions are suggested for the therapeutic purpose of diabetic muscle atrophy. Considering the proven safety of natural products, including medicinal plants, plant extracts or natural-product-derived compounds could be used for treating muscle atrophy as nutraceuticals.

Guava leaf (*Psidium guajava* L., GL) has been widely used as a functional tea due to its protective effect against hyperglycemia [11]. GL contains phenolic compounds such as gallic acid, epicatechin, quercetin, caffeic acid, and others [11]. These polyphenols have been shown to enhance muscle metabolism by improving glucose uptake, reducing oxidative stress, and modulating insulin signaling pathways, making GL extract (GLE) a promising candidate for metabolic health interventions [11]. However, despite polyphenols’ antioxidant and anti-inflammatory properties, polyphenols in GL exhibit limitations due to their poor bioavailability, which may affect their overall biological efficacy.

A recent study suggested that the bioconversion of natural products by lactic acid bacteria improved the bioavailability of active ingredients [12,13]. In addition, this process produces metabolites, which are considered key signaling mediators for a variety of physiological functions [14]. Although research investigating the effects of natural products bioconverted by microbial is limited, a previous study showed that the fermentation of red ginseng extract by *Lactobacillus plantarum* increased antioxidant effects [15]. Furthermore, the co-fermentation of *Bacillus* and *Monascus anka* released soluble phenolic compounds, which have higher biological activity [16]. These previous studies indicate that even though lactic acid bacteria and natural products have their own functional effects, when fermented together, their functionality is enhanced by the production of more active ingredients.

In this study, bioconverted GL (FGL) with *Lactobacillus plantarum* was treated to expect the ameliorative effect on muscle atrophy in T2DM mice.

In our previous study, bioconversion with LP led to a higher accumulation of hydroxyl-isocaproic acid (HICA) and hydroxyl-isovaleric acid (HIVA), which are known for their muscle-protective properties. Other previous studies have demonstrated that LP can enhance the bioavailability of flavonoids and phenolic acids through enzymatic modifications, thereby improving their functional effects [17,18]. Additionally, bioconverted GLE by *L. Firmentum* (LF) was more effective in glycemic control compared to LF in T2DM mice [19]. Notably, GFL further alleviates fatty liver by improving SIRT1-mediated energy metabolism. The result suggests that metabolites exclusive to GFL may contribute to this difference, leading to the superior effect of GFL compared to LF. Based on this evidence, this study hypothesized that bioconversion of GLE using LP would enhance its efficacy and mitigate muscle damage in T2DM by generating biologically active metabolites that regulate the muscle degradation pathway.

## 2. Results

### 2.1. Effects of FGL Treatment on Body Weight and Food Intake in T2DM Mice

After diabetes was induced, the body weight of the DMC group was significantly higher compared to that in the CON group. The skeletal muscle weight (g/kg) was decreased in the DMC group, but it was not normalized by FGL supplementation. In addition, body weight was not changed by FGL supplementation in T2DM mice. Food intake was reduced by FGL supplementation in T2DM mice (Table 1).

### 2.2. UHPLC-LTQ-Orbitrap-MS/MS Chromatogram of FGL

**Figure 1:**
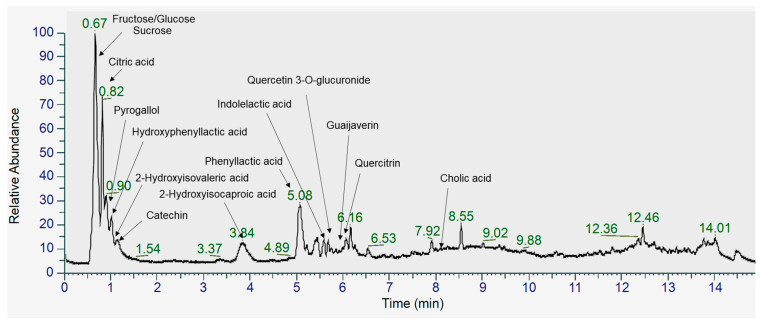
Total ion chromatogram (TIC) of metabolites identified in FGL using UHPLC-Orbitrap-MS/MS analysis in negative ionization mode.

FGL contains hydroxyisocaproic acid (HICA), hydroxyisovaleric acid (HIVA), cholic acid, hydroxyphenyllactic acid, quercetin 3-O-glucuronide, quercitrin, and catechin according to UHPLC-LTQ-Orbitrap/MS/MS analysis (Figure 1, Table 2).

### 2.3. Effects of FGL Treatment on Glycemic Regulation in T2DM Mice

**Figure 2:**
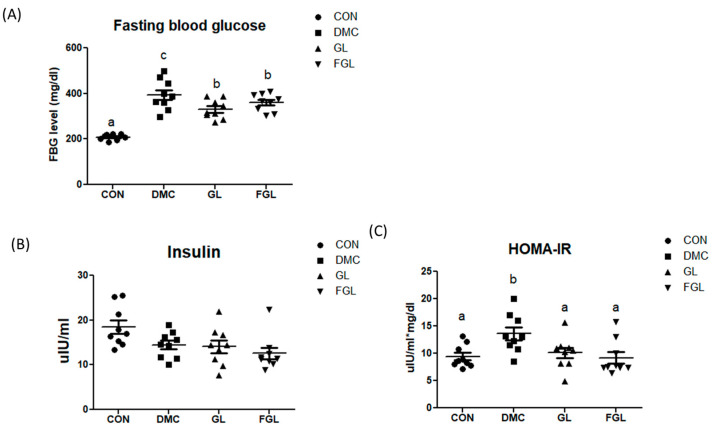
FGL supplementation regulated glycemic index in T2DM mice. (**A**) Fasting blood glucose, (**B**) insulin, and (**C**) HOMA-IR values are means ± SEM. Means with different letters were significantly different. (*p* < 0.05) n = 9 mice in each group. CON, normal mice (negative control); DMC, type 2 diabetic mice (positive control); GL, T2DM mice supplemented with low dose (100 mg/kg BW) of GLE; FGL, type 2 diabetic mice supplemented with bioconverted GLE (50 mg/kg BW) of GLE.

The final FBG level was significantly increased in the DMC group compared to that in the CON group. The FBG levels of GL and FGL groups showed significantly lower than the DMC group (Figure 2A).

Plasma insulin levels were not different among the groups. The homeostasis-model-assessment-estimated insulin resistance (HOMA-IR) was calculated to estimate insulin sensitivity by using the fasting plasma glucose and insulin concentrations. HOMA-IR in the DMC group was increased compared to that of the CON group. HOMA-IR was reduced by GL and FGL supplementation compared to the DMC group (Figure 2C).

### 2.4. Effects of FGL Treatment on Muscle Strength in T2DM Mice

**Figure 3:**
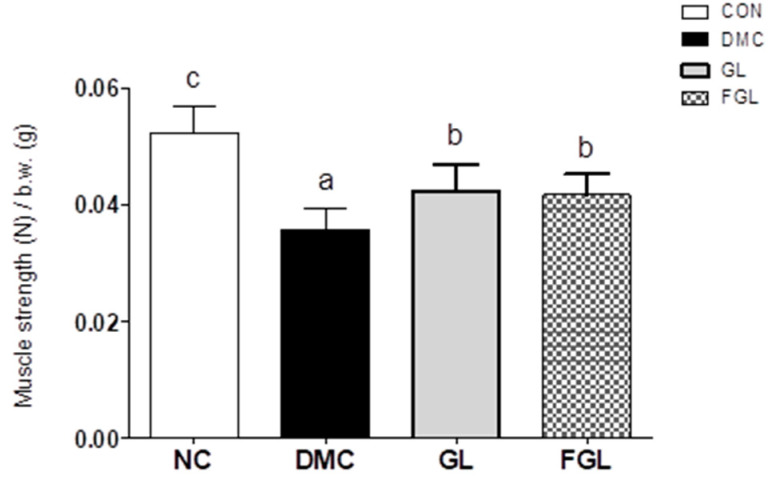
FGL supplementation improved muscle strength in T2DM mice. Values are means ± SEM. Means with different letters were significantly different. (*p* < 0.05) n = 9 mice in each group. CON, normal mice (negative control); DMC, type 2 diabetic mice (positive control); GL, T2DM mice supplemented with low dose (100 mg/kg BW) of GLE; FGL, type 2 diabetic mice supplemented with bioconverted GLE (50 mg/kg BW) of GLE.

Grip strength test was performed to evaluate muscle strength. Grip strength was decreased in the DMC group compared to the CON group. Muscle strength was enhanced by GL and FGL supplementation in T2DM mice (Figure 3).

### 2.5. Effects of FGL Treatment on Skeletal Muscle Morphology in T2DM Mice

**Figure 4:**
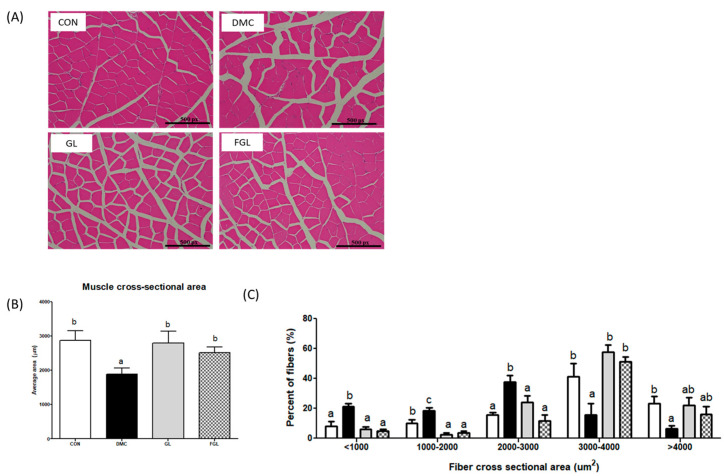
FGL supplementation enhanced skeletal muscle morphology (×200) in T2DM mice. (**A**) Representative images of fiber size alteration, (**B**) muscle fiber CSA in each group, and (**C**) frequency histograms showing the distribution of CSA of muscle fibers. Values are means ± SEM. Means with different letters were significantly different. (*p* < 0.05) n = 5 mice in each group. CON, normal mice (negative control); DMC, type 2 diabetic mice (positive control); GL, T2DM mice supplemented with low dose (100 mg/kg BW) of GLE; FGL, type 2 diabetic mice supplemented with bioconverted GLE (50 mg/kg BW) of GLE.

The skeletal muscle of diabetic mice showed an irregular fiber structure and reduced the skeletal muscle cross-section area (CSA) compared to those of the CON group. However, the skeletal muscle CSA was significantly increased by GL and FGL supplementation in T2DM mice (Figure 4). Additionally, fiber size distribution further showed that DMC increased the proportion of smaller fibers (<2000 µm^2^), while GL and FGL supplementation shifted the distribution toward larger fibers (3000–4000 µm^2^). These findings suggest that GL and FGL supplementation mitigated diabetes-induced muscle atrophy by improving overall fiber size and distribution.

### 2.6. Effects of FGL Treatment on Insulin-Signaling-Related Markers in T2DM Mice

**Figure 5:**
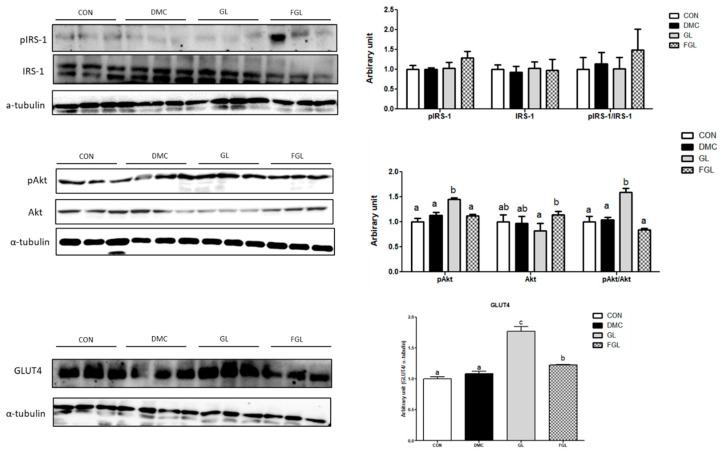
FGL supplementation enhanced insulin signaling pathway of skeletal muscle in T2DM mice. Values are means ± SEM. Means with different letters were significantly different. (*p* < 0.05) n = 6 mice in each group. CON, normal mice (negative control); DMC, type 2 diabetic mice (positive control); GL, T2DM mice supplemented with low dose (100 mg/kg BW) of GLE; FGL, type 2 diabetic mice supplemented with bioconverted GLE (50 mg/kg BW) of GLE.

The protein levels of pIRS-1 and IRS-1 were not different among the groups. The protein level of pAkt was increased in the GL group compared to that of the DMC group. The protein level of Akt was not changed by GL and FGL supplementation. The ratio of pAkt/Akt was increased in the GL group compared to that of the DMC group. The protein level of GLUT4 was increased in the GL group compared to that of the DMC group (Figure 5).

### 2.7. Effects of FGL Treatment on UPS-Related Markers in T2DM Mice

**Figure 6:**
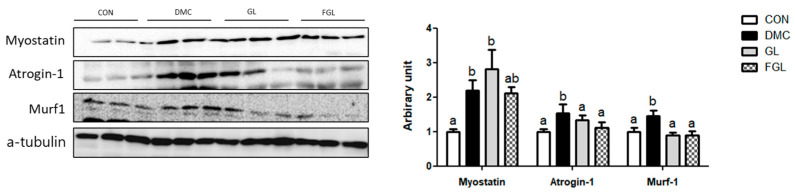
FGL supplementation reduced UPS of skeletal muscle in T2DM mice. Values are means ± SEM. Means with different letters were significantly different. (*p* < 0.05) n = 6 mice in each group. CON, normal mice (negative control); DMC, type 2 diabetic mice (positive control); GL, T2DM mice supplemented with low dose (100 mg/kg BW) of GLE; FGL, type 2 diabetic mice supplemented with bioconverted GLE (50 mg/kg BW) of GLE.

The protein levels of Myostatin, Atrogin-1, and Murf-1 were increased in the DMC group compared to those of CON group. The protein levels of Atrogin-1 and Murf-1 were decreased by GL and FGL supplementation in T2DM mice (Figure 6).

### 2.8. Effects of FGL Treatment on Apoptosis-Related Markers in T2DM Mice

The protein level of Bax was not different among the groups. The protein level of Bcl2 was increased in the GL and FGL groups compared to that of the DMC group. The ratio of Bax/Bcl2 was decreased in the FGL group compared to that of the DMC group.

**Figure 7:**
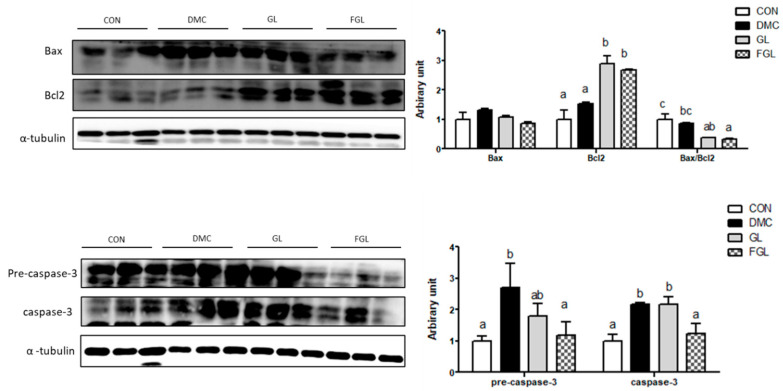
FGL supplementation suppressed excessive apoptosis of skeletal muscle in T2DM mice. Values are means ± SEM. Means with different letters were significantly different. (*p* < 0.05) n = 6 mice in each group. CON, normal mice (negative control); DMC, type 2 diabetic mice (positive control); GL, T2DM mice supplemented with low dose (100 mg/kg BW) of GLE; FGL, type 2 diabetic mice supplemented with bioconverted GLE (50 mg/kg BW) of GLE.

The protein levels of pre-caspase-3 and caspase-3 were increased in the DMC group compared to those of the CON group. The protein levels of pre-caspase-3 and caspase-3 were decreased by FGL supplementation compared to those of the DMC group (Figure 7).

### 2.9. Effects of FGL Treatment on mTOR-Autophagy-Related Markers in T2DM Mice

**Figure 8:**
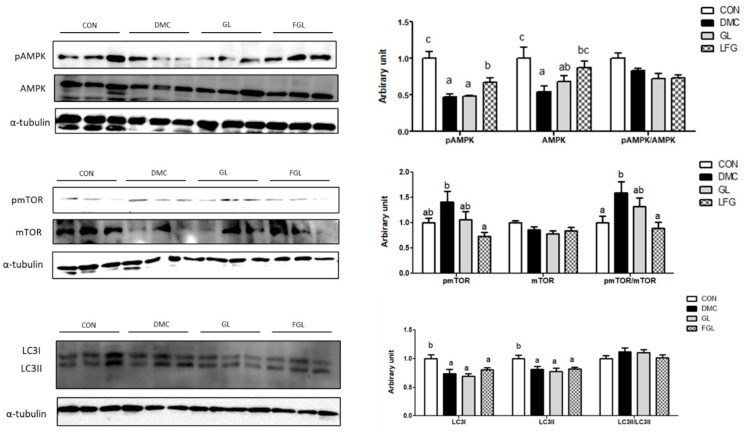
FGL supplementation enhanced mTOR-autophagy of skeletal muscle in T2DM mice. Values are means ± SEM. Means with different letters were significantly different. (*p* < 0.05) n = 6 mice in each group. CON, normal mice (negative control); DMC, type 2 diabetic mice (positive control); GL, T2DM mice supplemented with low dose (100 mg/kg BW) of GLE; FGL, type 2 diabetic mice supplemented with bioconverted GLE (50 mg/kg BW) of GLE.

The protein levels of pAMPK and AMPK were decreased in the DMC group compared to those of the CON group. The protein levels of pAMPK and AMPK were increased by FGL supplementation compared to those of the DMC group. The protein level of pmTOR and the ratio of pmTOR/mTOR were decreased by FGL treatment in T2DM mice. The protein levels of LC3I and LC3II were decreased in the DMC group compared to those of the CON group. The protein levels of LC3I and LC3II were not changed by FGL treatment compared to those of the DMC group (Figure 8).

### 2.10. Effects of FGL Treatment on Inflammation Related Markers in T2DM Mice

**Figure 9:**
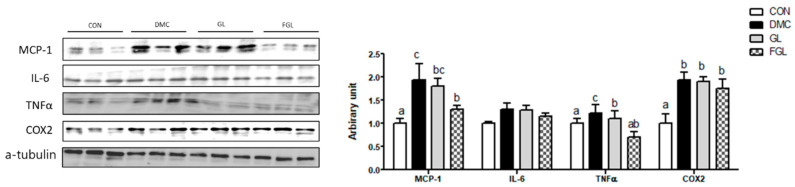
FGL supplementation attenuated inflammation of skeletal muscle in T2DM mice. Values are means ± SEM. Means with different letters were significantly different. (*p* < 0.05) n = 6 mice in each group. CON, normal mice (negative control); DMC, type 2 diabetic mice (positive control); GL, T2DM mice supplemented with low dose (100 mg/kg BW) of GLE; FGL, type 2 diabetic mice supplemented with bioconverted GLE (50 mg/kg BW) of GLE.

The protein levels of tumor necrosis factor alpha (TNFα), monocyte chemoattractant protein-1 (MCP-1), and cyclooxygenase (COX)2 were increased in the DMC group compared to those of the CON group. The protein level of TNFα in the GL group was decreased compared to the DMC group. The protein levels of MCP-1 and TNFα in FGL group were decreased compared to the DMC group (Figure 9).

## 3. Discussion

Skeletal muscle atrophy is one of the serious complications of diabetes and is known to gradually worsen as diabetes progresses [1,2]. Previous studies indicate that diabetic patients had a 10–11 times higher prevalence of muscle atrophy compared to non-diabetic patients [2,3]. As the prevalence of diabetic patient increases, the number of patients with diabetic muscle atrophy is expected to increase further in the future. Therefore, therapeutic strategies including nutritional intervention for muscle atrophy are needed.

It is widely known that probiotics can influence intestinal permeability as well as metabolic and signaling pathways, including systemic inflammation, energy, and glucose metabolism [20,21]. Evidence showed that L. *acidophilus*, L. *acidophilus La5*, and *Bifidobacterium animalis* subsp. *lactis Bb12* supplementation improved insulin sensitivity in many clinical studies [22,23,24]. LP treatment regulated body weight and blood glucose in animal models of obesity and diabetes [25]. In addition, LP HAC01 has an effect on hepatic glucose metabolism by increasing the phosphorylation of AMPK and Akt in a T2DM mouse model [26]. Moreover, the bioconversion of natural products by lactic acid bacteria produces metabolites, which regulate a variety of mechanisms. Metabolites derived from natural products and probiotics include short-chain fatty acids, peptides, phenolic acids, and flavonoids, which provide physiological benefits to the host. Although different studies have demonstrated the anti-diabetic effect of probiotics, the molecular mechanisms of metabolites derived from probiotics have not been fully investigated. Therefore, it is necessary to discover probiotic strains and natural products with functional properties specific to T2DM. In this study, GLE bioconverted by LP was treated to expect the ameliorative effect on muscle atrophy in T2DM mice. In our previous study, GLE bioconverted by LF showed a superior effect on hyperglycemia-induced hepatic damage compared to LF alone despite the very low dose of GLE used. This means that the bioconversion of natural products by lactic acid can be used effectively as a promising therapeutic agent.

The main metabolites derived from FGL are HICA, HIVA, hydroxyphenyllactic acid, quercetin 3-O-glucuronide, quercitrin, catechin, and cholic acid. In particular, HICA and HIVA are metabolites of the branched-chain amino acids (BCAAs) leucine and valine [27]. According to our previous study, the bioconversion of GLE with LP significantly increased these bioactive compounds compared to GLE, suggesting improved bioavailability and potential efficacy. BCAAs are known to have critical roles for skeletal muscle anabolism and energy homeostasis [28]. Impaired BCAA catabolism is associated with abnormal glucose utilization through insulin resistance, which is a major risk factor of muscle atrophy. In a recent study, HICA attenuated protein degradation by reducing inflammation in C2C12 cells [29]. Furthermore, chronic HICA treatment improved muscle recovery after immobilization-induced atrophy [28]. Although research on these metabolites is limited, based on previous studies, it can be assumed that these substances may help relieve muscle damage in diabetes.

Persistent hyperglycemia in diabetes can induce glucotoxicity and lipotoxicity through distinct mechanistic pathways, promoting muscle wasting. Therefore, appropriate glucose metabolism is important for skeletal muscle function. In this study, both FGL and GLE attenuated hyperglycemia, demonstrated by reduced FBC. In our previous study, GLE bioconverted by LF reduced FBG and HbA1c levels in T2DM mice [19]. It can be inferred that GLE bioconverted by both LF and LP produces more abundant flavonoids including catechin and quercetin, which are different from GLE alone, contributing to its glycemic control effect in diabetic mice through different mechanisms [30,31]. Although LF and LP produce different metabolite profiles during bioconversion, certain common bioactive metabolites derived from guava leaf fermentation—such as quercetin and cholic acid—are known to exert anti-diabetic effects, including the regulation of glucose metabolism and the improvement of insulin sensitivity. These metabolites may contribute to the beneficial effects observed in both studies.

In addition, cholic acid rich in FGL is known to improve glucose tolerance demonstrated by reduced FBG, HOMA-IR, and insulin level in T2DM mice [32]. Furthermore, a recent study showed that HICA and HIVA have the potential to influence glucose regulation by modulating the composition of the gut microbiome [33].

In the molecular level, GLE bioconverted by LF enhanced hepatic insulin signaling and energy metabolism in T2DM mice [19]. As a major tissue for glucose disposal, skeletal muscle also contributes to regulate glucose metabolism. In this study, FGL supplementation contributes to reduce FBG by the down-regulation of inflammation and apoptosis in diabetic skeletal muscle. On the other hand, GLE supplementation increased the protein level of pAkt and GLUT4 in skeletal muscle of T2DM mice. These results indicate that both GLE and FGL are beneficial for attenuating hyperglycemia in T2DM, but the effect may be mediated by different mechanisms resulting from the bioconversion of GLE. The differential effect of FGL on insulin signaling, despite its superior anti-inflammatory activity, may be attributed to the metabolic shift induced by bioconversion, which likely prioritizes muscle homeostasis through anti-catabolic pathways rather than directly augmenting insulin signaling. Furthermore, it is possible that certain insulin-sensitizing compounds are modified during fermentation. Additionally, substances in GFL have potential to improve the balance of intestinal microorganisms, thereby alleviating hyperglycemia.

In T2DM, fat accumulation promotes the production of inflammatory cytokines/chemokines that inhibit myogenesis and trigger muscle catabolism [34]. Among them, TNFα and MCP-1 are important mediators involved in catabolic responses and metabolic disturbances such as insulin resistance [35]. In this study, FGL treatment decreased the protein levels of inflammatory cytokine/chemokine (MCP-1 and TNFα) in the skeletal muscle of T2DM mice. In our previous study, GLE bioconverted by LF supplementation reduced hepatic inflammation by the regulation of NFkB and associated inflammatory cytokines [19]. The results demonstrated that bioconverted GLE by both LP and LF contains catechin and quercetin, which contribute to decrease inflammation in liver and skeletal muscle in T2DM mice. Furthermore, cholic acid present in FGL reduced inflammation in hepatic tissue during ischemia/reperfusion injury [36]. In particular, as an inflammatory mediator, cholic acid modulates intestinal permeability, which can also influence skeletal muscle metabolism and functionality [37]. It can be inferred that functional substances rich in FGL have an ameliorative effect on hyperglycemia-induced inflammation, which can lead to protein degradation in skeletal muscle.

Accordingly, we investigated whether the reduced inflammation by FGL prevents hyperglycemia-induced skeletal muscle atrophy. Atrogin-1 and Murf-1 play key roles in the development of skeletal muscle atrophy by promoting the protein degradation [3]. The suppression of Atrogin-1 and Murf-1 is known to ameliorate muscle atrophy in atrophic conditions [38]. Interestingly, both GLE and FGL supplementation decreased the protein levels of Atrogin-1 and Murf-1, indicating that they protect against hyperglycemia-induced skeletal muscle degradation. In line with this, GL and FGL supplementation prevented hyperglycemia-induced decreases in muscle strength and CSA in the skeletal muscle of T2DM mice. To further characterize these effects, we analyzed the CSA distribution of muscle fibers. In diabetic mice, there was a marked shift toward smaller fibers (<1000 μm^2^) and a significant reduction in larger fibers (>3000 μm^2^), indicating muscle atrophy and impaired regeneration. GL and FGL supplementation partially normalized this distribution, making it more comparable to that of the control group. Notably, both treatments decreased the proportion of small fibers and increased that of medium-to-large fibers (2000–4000 μm^2^), suggesting an attenuation of muscle damage under diabetic conditions. A previous study showed that HICA supplementation enhanced muscle function [39]. In the molecular level, HICA down-regulated protein degradation and the inflammation pathway in C2C12 cells [29]. Considering that muscle atrophy and inflammation exacerbate metabolic changes caused by T2DM, FGL might be useful in preventing T2DM-related metabolic disorders.

Moreover, autophagy deficiency in T2DM also contributes to muscle atrophy. It is widely known that insufficient autophagy leads to inefficient clearance of altered misfolded proteins, which can also lead to muscle damage [40]. On the other hand, excessive autophagy in cases of catabolic states contributes to a loss of skeletal muscle mass and function [41]. Abnormal autophagy increases dysfunctional mitochondria, the disorganization of sarcomere, and cell death, resulting in the degeneration of muscle fibers [42]. Therefore, restoring the baseline level of autophagy appears to be a potential therapeutic strategy to maintain muscle mass and function. In this context, we investigated mTOR-autophagy-related markers to demonstrate the effect of FGL on diabetic skeletal muscle. In particular, AMPK enhances autophagy by the inhibition of mTOR activation and increasing the expression of LC3II, which initiates the formation of autophagosome [43]. In this study, the mTOR-autophagy pathway in skeletal muscle was inhibited in T2DM mice. FGL supplementation contributed to normalizing the mTOR-autophagy-related pathway, demonstrated by an increased protein level of pAMPK and a reduced pmTOR/mTOR ratio in T2DM mice. However, the LC3-II levels remained unchanged in the current study despite improvements in upstream signaling. This may be due to the dynamic and transient nature of autophagy, which is highly dependent on timing. In our previous study, a higher dose (200 and 500 mg/kg) of GLE supplementation regulated the AMPK-mTOR pathway in dexamethasone-induced muscle atrophy mice [44]. This indicates that the bioconversion of GLE by LP was more effective against diabetic muscle atrophy compared to GLE, even with lower doses of GLE.

In addition, insufficient autophagy can promote the amplification of apoptotic signals [45]. Excessive apoptosis in skeletal muscle promotes protein degradation, which results in muscle damage. During apoptosis, proapoptotic Bax induces oligomerization, mitochondrial permeabilizing, and the release of cytochrome c into the cytoplasm [46]. Then, the activation of caspase family induces apoptosis [47]. Among them, caspase-3 is a frequently activated protease, which is triggered in cells by intrinsic and extrinsic pathways of apoptosis [48]. As an executioner caspase, caspase-3 induces apoptosis through the destruction of cellular structures [48]. In the current study, hyperglycemia-induced excessive apoptosis in T2DM mice. FGL supplementation reduced the Bax/Bcl2 ratio, which is a sensitive indicator for apoptosis. Furthermore, FGL supplementation reduced the protein levels of pre-caspase-3 and mature caspase-3 in T2DM mice. The result suggests that substances in FGL might protect myofibrils and control protein degradation by the normalization or inhibition of apoptosis. In our previous study, a high dose (500 mg/kg) of GLE supplementation reduced apoptosis in the muscle atrophy model [44]. The result suggests that GLE bioconvertion by LP produces different substances compared to GLE itself, even with a low dose of GLE. Furthermore, these substances have potential to prevent muscle degradation by the regulation of autophagy and apoptosis in T2DM mice.

In summary, both GLE and FGL supplementation regulated muscle strength, CSA, and UPS-related markers in T2DM mice. Furthermore, only FGL supplementation attenuated skeletal muscle inflammation and apoptosis with improved mTOR-autophagy-related pathway, which was not observed with GLE supplementation. Particularly, an enhanced anti-inflammatory response by FGL may primarily affect muscle preservation through the suppression of muscle catabolism rather than directly enhancing insulin signaling. The results demonstrated that although both GLE and FGL have beneficial effects on the strength and morphology of skeletal muscle, in a long-term perspective, FGL is expected to help alleviate inflammatory response and apoptosis that promotes the mechanisms underlying muscle atrophy.

The differential effect of FGL might be contributed to metabolites derived from the co-fermentation process of guava leaf and LP, including BCAA, their catabolites (HICA and HIVA), and organic acids, which exert additive and/or synergistic effects on diabetic muscle atrophy. Although FGL was administered at half the dose of GLE, it exhibited comparable or superior effects in several molecular markers, suggesting higher potency. It can be suggested that the bioconversion of GLE produced metabolites derived from GLE and LP, which can be considered an effective process to attenuate hyperglycemia-induced muscle atrophy in T2DM. However, the quantification of bioactive compounds is necessary to directly compare the efficacy of GLE and FGL under standardized conditions, thereby validating the observed potency differences. In addition, future studies using purified metabolites and pathway-specific inhibitors are needed to confirm the direct role of FGL-derived compounds, such as HICA and HIVA, in diabetic muscle atrophy. Moreover, further investigations are needed to determine whether these molecular alterations ultimately translate into clinically meaningful outcomes. In this study, LP-derived metabolites were not specifically examined due to the unclear effects observed in our previous study, which may slightly limit the distinction between bioconversion and probiotic effects. Hence, we focused on the bioconverted GLE as a potential agent for the treatment of diabetic muscle atrophy. It is meaningful that this study demonstrated the effects of FGL supplementation on hyperglycemia induced muscle atrophy for the first time. Furthermore, we expect that the effect of GFL may potentially be attributed to the improvement of the intestinal environment, although more specific mechanisms need to be investigated.

## 4. Materials and Methods

### 4.1. Preparation of Extract

Guava leaf was purchased in dried form (Goodherb, Dongdaemun-gu, Seoul, the Republic of Korea). Guava leaf was extracted twice in 50% ethanol at room temperature. The extract was prepared in powder form through solvent evaporation, freeze-drying, and powdering. The extraction’s yield was about 20.3%. The extract was dissolved in distilled water at a suitable concentration for the experimental design.

### 4.2. Lactobacillus Strains and Culture Conditions

*Lactiplantibacillus plantarum* KGMB 00831 (Korean Gut Microbiome Bank) was stored at −80 °C in de Man, Rogosa, and Sharpe (MRS) broth supplemented with 20% (*v*/*v*) glycerol. For activation, 0.1 mL of the bacterial stock was spread on MRS agar and incubated anaerobically at 37 °C for 24 h. Single colony was isolated through streaking and further cultured on MRS agar for 20 h at the same temperature. A single colony was then transferred into 5 mL of MRS broth in a 14 mL round-bottom tube (RB tube) and incubated for 18 h at 37 °C. The bacterial culture was adjusted to an optical density (OD) of 1.0 at 600 nm using a spectrophotometer, corresponding to approximately 2.02 × 10⁹ CFU/mL. These activated LAB cells were subsequently used in the bioconversion experiments. All cultures were maintained under anaerobic conditions in a chamber (Coy Laboratory Products, Grass Lake, MI, USA) with a gas mixture of 10% CO_2_, 5% H_2_, and 85% N_2_. *L. plantarum* was selected due to its well-documented enzymatic activity and metabolic versatility, which are advantageous for the bioconversion of plant-derived compounds.

### 4.3. Bioconversion of Guava Leaf Extract

The bioconversion method of guava leaf was conducted according to previous research [13]. Briefly, guava leaf extract was added to MRS broth at a final concentration of 0.5% (*w*/*v*) in 50 mL conical tube containing 15 mL of medium. The mixture media were sterilized by autoclaving at 121 °C for 15 min and filtered through a 0.22 μm polytetrafluoroethylene (PTFE) filter. The bacterial culture, adjusted to OD 1.0, was inoculated into the sterilized media at 5% (*v*/*v*) in triplicate.

Samples were harvested after 24 h of incubation at 37 °C, corresponding to the mid-stationary phase. The cultures were centrifuged at 11,001× *g* for 10 min at 4 °C. For metabolite extractions, 8 mL of 100% MeOH was added to 4 mL of the filtered (0.22 μm) supernatant, and the mixture was incubated at 200 rpm for 2 h in a rotary shaker. After incubation, 10 mL of extracts from each biological replicate was pooled and dried using a speed vacuum concentrator. The resulting dried metabolite extract was used for metabolite analysis and bioactivity evaluation in an in vivo model.

### 4.4. UHPLC-LTQ-Orbitrap-MS/MS Analysis of FGL

The methodology was performed as described in our previous study [13]. Briefly, dried extract was dissolved in 50% methanol and filtered through 0.2 μm polytetrafluoroethylene filter. GFL underwent ultrahigh-performance liquid chromatography and mass spectrometry (UHPLC-LTQ-Orbitrap-MS/MS) analysis using a UHPLC system equipped with a Vanquish binary pump H system (Thermo Fisher Scientific, Waltham, MA, USA) equipped with an autosampler and a Phenomenex KINETEX^®^ C18 column (100 mm × 2.1 mm, 1.7 μm) (Phenomenex, Torrance, CA, USA).

### 4.5. Animal and Experimental Design

Four-weeks-old male C57BL/6 mice (Raon Bio, Yongin-si, Gyeonggi-do, the Republic of Korea) were housed under controlled temperature (22 ± 1 °C), humidity (50 ± 5%), and 12 h light and dark cycle. All mice were first randomly divided into two groups: the control group that was fed a 10% kcal control diet (D12450J; matching sucrose to D12492, Research Diets, New Brunswick, NJ, USA) and the type 2 diabetes mellitus (T2DM) group, which was fed a 60% kcal high-fat diet (HFD) (D12492; Research Diets, New Brunswick, NJ, USA). After continuous HFD feeding for 4 weeks, mice were injected with 60 mg/kg streptozotocin solution (STZ, dissolved in a citric acid buffer, 0.01 M, pH 4.5) once a week for 2 consecutive weeks [19,49].

Every week after the second injection, fasting blood glucose (FBG) was detected using a glucose meter (LifeScam Omc., Milpitas, CA, USA). Mice with FBG > 300 mg/dL at least twice in four weeks were judged as diabetic model mice. After 6 weeks of diabetes induction, the animals were divided into 4 experimental groups as follows: CON: normal control group, DMC: diabetes mellitus control group, GL: diabetic mice supplemented with GLE (100 mg/kg BW), FGL: diabetic mice supplemented bioconverted GLE (50 mg/kg BW) (n = 9 per group) [18]. GL and FGL extracts were suspended in distilled water and orally gavaged every day for 14 weeks. Body weight, food intake, and FBG level were measured once a week. All experiments were approved by Kyung Hee University for animal welfare [KHSASP-20-060] and were performed in accordance with the guidelines.

### 4.6. Grip Strength Test

A grip strength meter (grip test package GS3 (25N); Harvard Apparatus, Holliston, MA, USA) was used to measure forelimb grip strength. The maximum force of a mouse’s grip on a metal bar (N) was recorded. The average of 5 trials was used for analysis [50].

### 4.7. Histological Analysis

Skeletal muscle (gastrocnemius) was paraffin-embedded and sectioned at 4 µm-thickness. The sections were stained with hematoxylin and eosin to evaluate the histological changes in skeletal muscle tissue. The average CSA (%) of skeletal muscle (gastrocnemius) was analyzed using an Image J software (version1.54, NIH, Bethesda, MD, USA) [51].

### 4.8. Protein Extraction and Western Blot Analysis

Skeletal muscle (gastrocnemius) was used to analyze Western blot. In total, 30 μg of protein for each group was used for Western blot analysis. The total tissue proteins (n = 6) were separated by 8~12% SDS-PAGE gel electrophoresis and then transferred to polyvinylidene fluoride membrane (Millipore, Billerica, MA, USA). Then, incubated overnight with primary antibodies and probed with respective secondary antibodies. The protein bands were visualized by using the enhanced chemiluminescence (ECL) luminol reagent (Biorad, Hercules, CA, USA). All quantifications were normalized to the levels of α-tubulin (cytosol) [52].

The following primary antibodies were used: p-IRS-1 (Ser302) (#2381), IRS-1 (#2382), MCP-1 (#2029), pmTOR (#2971), mTOR (#2972), LC3 (#2775), pAMPK (#2535), AMPK (#2532), COX2 (#12282), tumor necrosis factor-α (TNF-α) (#11948), IL-6 (#12912), caspase-3 (#9662), Bcl2 (#3498), Bax (#2772), p-Akt (Ser473) (#4058), Akt (#4691), SIRT1 (#2028) (Cell Signaling Technology, Danvers, MA, USA, 1:200), PGC-1α (sc-517380), Atrogin-1 (sc-166806), Murf-1 (sc-398608), GLUT4 (sc-53566) (Santa Cruz Biotechnology, Santa Cruz, CA, USA, 1:200), myostatin (ab124721) (Abcam, Cambridge, Cambridgeshire, UK, 1:2000), and α-tubulin (Sigma Aldrich, St. Louis, MO, USA, 1:5000).

### 4.9. Statistical Analysis

All data were presented as means ± SEM. The significance of differences was analyzed by one-way ANOVA with post hoc Duncan multiple range test. Outliers were assessed using SPSS’s Explore procedure, with boxplots and IQR-based detection applied separately to each group. A probability level of *p* < 0.05 was considered statistically significant. All statistical analysis was using SPSS software (version 23.0 for Windows, SPSS Inc., Chicago, IL, USA).

## 5. Conclusions

Taken together, the present study showed that FGL has superior efficacy compared to GLE in hyperglycemia-induced muscle atrophy by the inhibition of the inflammation and catabolic pathways in T2DM mice. To our knowledge, this is the first study to demonstrate the protective effects of GLE bioconverted by LP on skeletal muscle under diabetic conditions. In conclusion, these findings provide promising preclinical evidence supporting the potential of FGL as a nutraceutical candidate for managing diabetic muscle atrophy; however, further studies, including clinical trials, are required to validate its efficacy and safety in humans.

## Figures and Tables

**Table 1:** Effects of GFL supplementation on body weight, body composition, food intake, and liver weight in diabetic mice.

GROUP	CON	DMC	GL	FGL
Body weight (g)				
Before treatment	30.30 ± 0.75 ^a^	36.14 ± 0.87 ^b^	35.02 ± 0.69 ^b^	35.39 ± 0.74 ^b^
After treatment	30.71 ± 0.79 ^a^	43.51 ± 1.43 ^b^	40.21 ± 0.69 ^b^	41.72 ± 1.39 ^b^
Gain	0.41 ± 0.31 ^a^	7.38 ± 0.70 ^c^	5.19 ± 0.42 ^b^	6.34 ± 0.76 ^bc^
Muscle weight (g/kg BW)	0.54 ± 0.01 ^b^	0.40 ± 0.01 ^a^	0.43 ± 0.01 ^a^	0.42 ± 0.02 ^a^
Food intake (g/day)	2.97 ± 0.03 ^c^	2.65 ± 0.05 ^b^	2.51 ± 0.09 ^ab^	2.47 ±0.04 ^a^

Values are means ± SEM. Means with different letters were significantly different. (*p* < 0.05) n = 9 mice in each group. CON, normal mice (negative control); DMC, type 2 diabetic mice (positive control); GL, T2DM mice supplemented with GLE (100 mg/kg BW); FGL, type 2 diabetic mice supplemented with FGL (50 mg/kg BW).

**Table 2:** Identification of metabolites in FGL using UHPLC-Orbitrap-MS/MS analysis.

No.	Tentative Identification	RT (min) ^a^	Measured Mass (*m*/*z*)		Molecular Formula	Fragment Pattern	Δ ppm	DB ^b^
			[M−H]^−^	[M+H]^+^				
	Sugars							
1	Fructose/glucose	0.64	179.0564	-	C6H12O6	(−) 59 89 161 71 131	1.34	Web DB
2	Sucrose	0.63	341.1088	343.123	C12H22O11	(−) 89 101 119 179	0.5	Web DB
	Organic Acids							
3	Citric acid	0.82	191.0197	193.0343	C6H8O7	(−) 111 87 85 129	0.10	In-house DB
4	Hydroxyphenyllactic acid	1.03	181.0509	183.0651	C9H10O4	(−) 163 135 119 72	1.33	In-house DB
5	Phenyllactic acid	5.08	165.0559	-	C9H10O3	(−) 147 119 72 103	0.97	In-house DB
	Fatty Acids							
6	2-hydroxyisovaleric acid	0.64	117.0561	-	C5H10O3	(−) 71 99	3.13	In-house DB
7	2-hydroxyisocaproic acid	3.83	131.0716	-	C6H12O3	(−) 85 69	1.73	In-house DB
8	Cholic acid	8.02	407.2805	-	C24H40O5	(−) 425 408 283	3.23	In-house DB
	Flavonoids							
9	Catechin	1.17	289.0721	291.086	C15H14O6	(−) 245 125 203 109 179	1.32	In-house DB
10	Quercetin 3-O-glucuronide	5.68	477.0676	479.0814	C21H18O13	(−) 301 178 151 113	0.17	Web DB
11	Guaijaverin	5.93	433.0779	435.0916	C20H18O11	(−) 300 301 178 151 271	0.71	Web DB
12	Quercitrin	6.06	447.0934	449.1069	C21H20O11	(+) 303 345 85 86 129	0.43	In-house DB
	Etc.							
13	Pyrogallol	0.92	125.0247	-	C6H6O3	(−) 81 97 69 107	2.29	Web DB
14	Indolelactic acid	5.6	204.0669	206.0809	C11H11NO3	(−) 158 186 116 142	0.99	In-house DB

^a^ RT, retention time. ^b^ DB, database.

## Data Availability

The data presented in this study are available on request from the corresponding author. The data are not publicly available due to privacy. The studies do not involve humans.

## Abbreviations

Atrogin-1	Muscle atrophy F-box
BCAA	Branched-chain amino acids
BW	Body weight
CON	Control group
CSA	Cross-sectional area
DMC	Diabetes mellitus control
FBG	Fasting blood glucose
FGL	Fermented guava leaf
GL	Guava leaf (*Psidium guajava* L.)
GLE	Guava leaf extract
HICA	2-hydroxyisovaleric acid
HIVA	Hydroxyphenyllactic acid
HOMA-IR	Homeostasis-model-assessment-estimated insulin resistance
LF	*Lactobacillus Firmentum*
LP	*Lactobacillus plantarum*
mTOR	Mammalian target of rapamycin
Murf-1	Muscle ring finger-1
T2DM	Type 2 diabetes mellitus
UPS	Ubiquitin–proteasome system
